# Interested consumers’ awareness of harmful chemicals in everyday products

**DOI:** 10.1186/s12302-017-0127-8

**Published:** 2017-11-21

**Authors:** Sabrina Hartmann, Ursula Klaschka

**Affiliations:** 1 University Ulm, Albert-Einstein-Allee 11, 89081 Ulm, Germany; 2 University of Applied Sciences Ulm, Prittwitzstr. 10, 89075 Ulm, Germany

**Keywords:** Hazardous substances, Harmful substances, Risk communication and comprehension, Consumer products, Consumers’ attitudes, Consumers’ awareness

## Abstract

**Background:**

Everyday products can contain a multitude of harmful substances unnoticed by most consumers, because established risk communication channels reach only part of the society. The question is, whether at least interested and informed consumers are able to use risk communication tools and assess harmful chemicals in products.

**Results:**

An online survey investigated the awareness of 1030 consumers on harmful substances in everyday items. Participating consumers’ education level, knowledge in chemistry, and motivation were above society’s average. Although a large number of responses showed that survey participants were familiar with several aspects of the issue, the results revealed that knowledge in chemistry helped, but was not enough. Many participants assumed that products with an eco-label, natural personal care products, products without hazard pictograms or products produced in the European Union would not contain harmful substances. Most participants indicated to use hazard pictograms, information on the packaging, reports in the media, and environmental and consumer organizations as information sources, while information by authorities and manufacturers were not named frequently and did not receive high confidence. Smartphone applications were not indicated by many participants as information sources. The information sources most trusted were environmental and consumer organizations, hazard pictograms, and lists of ingredients on the containers. The declared confidence in certain risk communication instruments did not always correspond to the use frequencies indicated. Nearly all participants considered legislators as responsible for the reduction of harmful substances in consumer products.

**Conclusions:**

Misconceptions about harmful substances in products can be dangerous for the personal health and the environment. The survey indicates that motivation, educational level, and chemical expertise do not automatically provide an appropriate understanding of harmful substances in products. If well-informed consumers are not sufficiently capable to use risk information elements as revealed in this study, then this will be even more the case for the general public. Consumer awareness should be stipulated by an improved information strategy about chemical risks in consumer products with an extensive participation of the target groups and by more efforts by authorities and manufactures to build trust and to provide easily understandable information.

**Electronic supplementary material:**

The online version of this article (10.1186/s12302-017-0127-8) contains supplementary material, which is available to authorized users.

## Background

Modern society is experiencing a period of unprecedented consumption with an overwhelming multitude of chemical substances being used in consumer articles and commercial mixtures. Many substances classified as hazardous according to the EU regulation on classification and labeling (CLP Regulation) [1] are present in everyday products as regular ingredients, like, for example, preservatives in washing and cleaning agents, fragrances in personal care products, per- and polyfluorinated chemicals used in textile finishing, plasticizers in plastic materials, or heavy metals in electronic appliances. Many of these substances remain unnoticed by the average end-user who takes the benefits of the chemical constituents for granted and trusts that unwanted properties for man and the environment are negligible.

Risk communication provisions, such as hazard pictograms on the product containers, are established to aid consumers and workers to be aware of hazards and to implement a suitable risk management behavior so as to minimize exposure and hence risk. The understanding of the risk communication message by the recipients is one of the basic pillars of chemical legislation [2]. Risk communication is an important first step, but there are various indications that risk communication measures are not always as effective as intended, because they are not always understood in the way expected by the decision makers and are thus not sufficiently protective under the consumer and the environmental perspectives. Previous studies that evaluated the efficiency and effectiveness of risk communication yielded remarkable results: A large number of users in European and non-European countries struggled to understand ingredient lists and labels [3, 4]. Other studies analyzed the understanding of hazard pictograms and showed that end-users did not understand the signs correctly [5, 6]. Even correctly understood risk information did not necessarily lead to the intended risk reduction behaviors [5]. It was also described that illiterate persons had great difficulty to understand pictorial label information and safety instructions [6]. A European survey [7] where citizens should indicate whether they thought that certain products contained ‘chemical substances’ in general showed that it is also worthwhile to ask very simple and basic questions which do not require any previous knowledge. Participants of this survey were not asked about harmful substances but only about chemical ingredients. For a chemist the results of this survey were shocking because large numbers of participants in some EU member countries assumed that products like cleaning products (up to 9% of the participants in one EU member country), paint (up to 10%), electronic appliances (up to 37%), or furniture (up to 38%) would not contain chemical substances at all. Such results suggest that these citizens might have problems understanding risk communication tools. However, it is not certain that experts judge risks correctly [8, 9].

The hypothesis of the present study was, that understanding risk communication tools is a challenge even for interested and informed consumers. If even well-informed consumers were not sufficiently capable to use risk information elements, then this would be even more the case for the average population. A survey was conducted using an on line questionnaire with voluntary participants who represented a ‘best-case’ scenario, as nearly all of them indicated that they were interested in chemicals in everyday products and the majority had a higher education level and good self-reported chemical expertise. Thus, the motivation to observe and the capability to comprehend risk communication elements in this group is assumed to be higher than in the average population.

The questions focused on the following topics:

- Interest in and awareness of substances that are harmful for human health or the environment in consumer products.
- Knowledge about risk communication tools and confidence in the risk communication information.
- Attitude toward the responsibility for harmful substances in consumer products.
- Description of own risk-mitigation behavior.

The present survey is much more comprehensive than the two questions posed by the European Chemicals Agency (ECHA) on this subject in 2016 [10]. It can be seen as part of the evaluation and review of risk communication elements as laid down in step 4 of the recommendations set up by the ECHA [11]. The outcome of this survey contributes to develop improvements for effective risk communication within the European regulatory framework and contributes to the aim of a non-toxic environment by 2018 [12] and to the fitness check of the EU Chemicals legislation [13, 14].

In fact, products made in the European Union contain harmful substances as illustrated in Box 1.

### Box 1: Overview on information sources about harmful substances in products relevant for the present survey

There is a multitude of various provisions and labels for harmful chemicals depending on the types of consumer products. Some of these information sources are legally binding and must be provided by producers whereas other information is voluntary. The multitude of regulations and provisions, the multitude of potential harmful ingredients make hazard information a difficult and complex task (e.g., [15]), especially as legislation is not always coherent (e.g., [16]).

For example, single substances and mixtures come under the CLP Regulation [1] and should be classified and labeled with hazard pictograms if they contain hazardous substances above the respective thresholds for labeling. However, there are many everyday products which may contain harmful substances, and the consumer is not informed about their presence by hazard pictograms. For example, personal care products [17], food and pharmaceuticals are mixtures that are exempted from the CLP Regulation. Also articles (such as electronic products, textiles or articles of plastic materials) do not need to be labeled with hazard pictograms according to the CLP Regulation. Lists of ingredients are legally required on personal care products [18], washing and cleaning agents [19], pharmaceuticals, and prepackaged food. Even experts need time and knowledge to discern which of the ingredients in these lists are harmful for human health or the environment [4]. Ingredient lists are an important tool for toxicologists and dermatologists. However, patients who need to avoid specific allergens do not rely exclusively on ingredient labels. Surveys with patients suffering from fragrance allergy showed that the majority used the ingredient lists, but followed also ‘trial and error’ to find the products they could tolerate [20, 21]. Furthermore, there are various studies showing that the ingredient lists were not always correct [22–26].

Apart from hazard pictograms or the ingredient lists, legally required information on the packaging are, for example, labels for electric and electronic articles [27]. The construction products Regulation [28] foresees an indication of substances of very high concern in the declaration of performance for construction products. Furthermore, harmful chemicals are regulated in the toy safety Directive [29], in the Directive regarding medical devices [30], in the Directive regarding active implantable medical devices [31], in the Directive regarding in vitro diagnostic medical devices [32], and in the packaging Directive [33]. Toys for children usually fulfill stricter criteria concerning harmful substances than most other articles, but they are not necessarily free of them [29]. Products for children like textiles or personal care products are not liable to fulfill stricter standards compared to products for adults, and hence they can contain harmful substances. There are legal provisions for medical devices [30–32]; however, they might contain harmful substances at higher concentrations compared to everyday products, because their applications are often exempted from the REACH [34] and CLP provisions.

According to the REACH Regulation [34] consumers have the right to know whether an article contains the ‘substances of very high concern’, which are carcinogenic, mutagenic, toxic for reproduction, or very critical for the environment. It does not have to be indicated on a product whether it contains these substances, but every end-user has the right to send an inquiry for an article of interest to the manufacturer or the distributor who is liable to respond within 45 days [35, 36].

Products which are not compliant with legal requirements and which pose a serious risk to health and safety of consumers are notified in the European rapid alert system for products, called RAPEX. The essential data on products with serious risk are notified weekly in the on line public European rapid alert database [37, 38]. Furthermore, there are presumably non-compliant products which were not detected by surveillance activities which can contain higher levels of harmful substances than allowed.

Voluntary national or international product information of high quality standard are eco-labels such as the EU label for organic food [39], the EU ecolabel for non-food products [40] or the German Blue Angel [41]. However, these products are not necessarily free of harmful substances. Organic food produced according to the EU Regulation for organic food [39] or untreated food taken from the own private garden should not contain contaminations by synthetic pesticides or substantial environmental contaminations. Such food might contain natural harmful substances produced by the crop plants (such as alkaloids in nightshades or hydrogen cyanide in bitter almonds), contaminants resulting from the growth environment, from the production process or from storage (such as mycotoxins). Non-food-products with an ecolabel, for example, the Blue Angel [41], fulfill strict criteria, but they are not necessarily free of harmful substances.

Most natural personal care products contain natural hazardous substances [42].

Homeopathic products usually contain active ingredients, which can be highly toxic such as e.g., *Atropa belladonna*, arsenic or mercury that are more or less diluted in substances like water or sugar. Therefore, homeopathic products of low dilutions (‘low potencies’) can contain considerable and sometimes even lethal amounts of harmful substances [43], while high dilutions (‘high potencies’) might contain only very few or no molecules of the diluted substance. In addition, they do not need to undergo standardized official drug trials like conventional pharmaceuticals, hence their precise health effects are not always known [44–46].

Many natural pharmaceuticals are effective because of their content in natural harmful substances, e.g., eucalyptus oil, chamomile oil or peppermint oil.

It must be noted that the declaration of the safety of a product by the producer does not preclude the presence of harmful substances. Producers of personal care products or homeopathic medicine state that their products were safe, knowing that the products can contain harmful ingredients in various amounts.

Private information programs are, for example, the Oekotex standard for textiles [47], independent product tests [e.g., Oekotest [48] or information provided by the producer (legally required or as more or less informative voluntary advertisement)]. The smartphone application ToxFox [49] provides information about the presence of endocrine disruptors in personal care products and about SVHCs in articles. The smartphone application and the homepage CodeCheck [50] offer information about a multitude of various products based on data entered by consumers. The smell of a product can indicate volatile odorous compounds, such as solvents, fragrances or else, but is not generally an indication of harmful substances. People who indicate ‘own experiences’ as means to find out whether a product contains harmful substances, might run a high risk, because some effects are not reversible and others are long-term effects without immediate warning symptoms.

## Methods

The online survey was executed using the cloud-based software provided by the service company SurveyMonkey (http://www.SurveyMonkey.com). It was accessible in the internet between September 13 and October 31, 2016. The questionnaire was distributed in German language (The English text of the questionnaire is in the “Appendix”, the original German version in the Additional file 1). The questionnaire was sent to colleagues and friends with the appeal to distribute it further. The following online platforms and institutions got on board: on line newsletter of the German Chemical Society (http://www.gdch.de), the homepage of the science magazine Odysso on the German public television channel (http://www.swr.de/odysso/), employees of the German Environment Agency, the staff of the University of Applied Sciences Ulm, the mailing network on sustainability run by the universities of applied sciences in Baden-Wuerttemberg, the regional group of Friends of the Earth in Ulm, and the local nature conservation group GAU (http://www.gau.neu-ulm.de). The focus was on the protection of consumers and the environment, while risk communication at the workplace was not addressed. Participants responded to a maximum of 38 questions, with various branch points. Answers given to questions 15–27 will be analyzed in a separate study. All information collected was self-reported by the participants. Several questions offered the option to add free text. These comments were analyzed qualitatively.

In this study, consumer products comprise articles in terms of the European Chemicals Regulation (REACH) [34], mixtures like washing and cleaning agents or paints in the sense of the Regulation on Classification, Labelling and Packaging (CLP) [1], or any other consumer product, like food or pharmaceuticals. A hazard is defined as ‘a possible source of danger’ [11]. Hazardous substances are defined by standardized classification and labeling according to the criteria of the CLP Regulation [1]. Harmful substances are hazardous substances or substances of very high concern according to the REACH Regulation [34].

The data we received from the software provider comprised the numbers of responses and the date of the participants’ online access. The demographic information collected did not allow for the identification of any participant of the survey. The correlations between the responses and the demographic details of the participants were calculated using the software application ‘matrix laboratory’ (MATLAB) (https://www.mathworks.com). The age group below 20 years with only 25 respondents was too small for separate statistical analysis. Pearson’s *χ* ^2^ test was used to detect correlations for data sets containing nominally scaled variables. *p* values below 0.05 were considered significant.

## Results

### Interested survey participants with a high number of chemists and graduates

More than thousand individuals took part in the survey (Table 1). Most participants responded to all questions (1030 out of 1321 who had started in the beginning). The objective of the survey was to obtain a picture of the opinions of ‘best-case’ citizens who had a great interest in chemical risk communication and who were to a higher degree experts in this field than the average population. This was achieved by the distribution of the on line questionnaire via the snowball system. The study participants’ demography showed that the study group was far from being average:

**Table 1:** Study participants demography (*N* = total number of participants = 1030)

	Number (% of all participants)
Gender	
Female	615 (59.7)
Male	415 (40.3)
Parents of minor children	285 (27.7)
Educational level	
Presently at school	6 (0.6)
Presently student or in job training	107 (10.4)
Completed vocational training/apprenticeship	134 (13.0)
Foreman/business administrator	41 (4.0)
University degree/Ph.D.	713 (69.2)
Other	29 (2.8)
Working with chemicals or REACH	353 (34.3)
Member of an environmental organization	270 (26.2)
Member of a consumer organization	37 (3.6)
EU citizen	1004 (97.5)
Chemical intolerance of study participant or family member	255 (24.8)

Nearly all participants (98%) indicated that they were interested in chemicals which are harmful for human health or the environment and used in everyday products. The answers given indicated that participants took great care when going through the questionnaire. The strong commitment of the participants was also reflected in the large number of annotations made (402 notes in 11 open questions by 232 participants).

In total, 61% of the participants reported that their knowledge of chemistry was good or very good (Additional file 2: Table S1). Many young people (aged 20–29) in this survey indicated to be very good in chemistry (female: 42.3%, male 24.5%). Women from 60 years up reported most often to have no or little knowledge in chemistry (60–69: 63.8%, > 70: 78.6%). Of all the participants 34.3% declared that they were dealing with chemicals or REACH [34] at their workplace. These demographic data showed the high number of informed persons representing the intended ‘best-case’ scenario.

The percentage of participants with an academic degree (69.2%) was much larger than average in Germany (16% [51]). Three quarters (76%, *χ* ^2^ = 95.8, *p* < 0.0000) of persons with self-reported good and very good knowledge in chemistry have an academic career. More men (78%) had an academic career, compared to 63% of the women (*χ* ^2^ = 40.0, *p* < 0.000). Not everybody who is dealing with chemicals or REACH at the workplace (91%, *χ* ^2^ = 340.6, *p* < 0.0000) indicated to have good or very good knowledge in chemistry.

More than a quarter of the participants (26.2%) were members of an environmental organization. The percentages increased with age (20–29: 13%, 30–39: 25%, 40–49: 28%, 50–59: 34%, 60–69: 35%, ≥ 70: 39%) (*χ* ^2^ = 41.9, *p* < 0.0000). The shares of persons who were members in environmental organizations was equal for both sexes. (For comparison: the four important environmental organizations in Germany (Friends of the Earth Germany, Nature and Biodiversity Conservation Union, World Wide Fund for Nature, and Greenpeace Germany have more than 500,000 members each [52–55], which would be around 0.5% of the German population). A small fraction of the participants in the present survey (3.6%) were members of a consumer organization. (For comparison, the German Allergy and Asthma Organization (DAAB) [56] has 18,000 members and Foodwatch 35,000 [57].) There were participants (2.5%) who indicated to be members in consumer organizations as well as in environmental organizations.

The percentage of participants who had minor children (27.7%) was higher than the German average (17.6% [51]).

Almost a quarter of the participants (24.8%) indicated that they themselves or a family member suffered from chemical intolerance with a slight maximum in the age groups between 50 and 69 (by comparison around 30% of the German population suffer from allergies (e.g., contact allergy, food intolerance or respiratory diseases [58]).

More women (59.7%) participated in the survey than men (percentage of women in Germany 50.7% [51]). However, in the age groups above 60 years nearly twice as many men participated than women.

All age group were represented, but people under the age of 20 (2%) and over the age of 70 (5%) were less represented than the other age groups. Nearly every second participant (48%) was up to 40 years old. (The average age in Germany is 44 years [51].)

### Harmful substances unnoticed in consumer products

Participants were asked whether they knew that products can contain chemicals harmful for human health (question 6). Nearly 10% of the survey participants answered that they would not know this. Nearly half of these persons were between 20 and 39 years old or had an academic career. The answers depended on the knowledge in chemistry. More people with no or little chemical expertise (14.2%, *χ* ^2^ = 18.8, *p* < 0.0000) chose this option, but also 6.2% of persons who indicated to have a good or very good chemical expertise did not know that products can contain chemicals harmful for human health. Interestingly, 11 persons (*χ* ^2^ = 24.9, *p* < 0.0000) who are working with chemicals or REACH in their jobs selected this option.

Survey participants were also asked whether they knew that products could contain chemicals that are harmful for the environment (question 7). In all demographic groups, the number of participants who were familiar with this fact was higher than the number of persons who were aware of chemicals that are harmful for human health. Nevertheless, 6.6% responded that they did not know this.

### What do participants care about?

#### Strong interest in human health and environmental effects

**Fig. 1:**
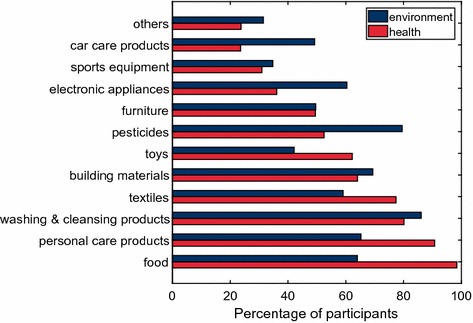
Respondents interested in product groups for human health reasons (red) or for environmental reasons (blue)

Survey participants could select product groups which were of interest to them for human health reasons (questions 3) or for environmental reasons (questions 4) (Fig. 1). Products with direct contact to the human body (food, personal care products, textiles, and toys) were selected more often because of human health interest, while products which are discharged mainly to the environment or are employed outdoor (such as pesticides and car care products) were selected more often for environmental reasons. Also individuals with little or no self-reported chemical expertise had these preferences. Washing and cleansing products, building materials, and furniture were selected as frequently for human health reasons as for environmental reasons. More than a quarter of the respondents (28.8%) were interested in pharmaceuticals for environmental reasons. Interestingly, 29.5% participants decided that all products were interesting for environmental reasons, while only 19.5% of the participants decided that all products were interesting for human health reasons.

All women were interested in personal care products for human health reasons (100%) compared to 86.3% of the participating men (*χ* ^2^ = 66.0, *p* < 0.0000). Most of the parents with minor children were interested for health reasons in toys (94.0%), compared to only 56.6% of persons without minor children (*χ* ^2^ = 130.3, *p* < 0.0000).

#### Personal health and responsibility for future generations as major motives

Survey participants could choose between seven options as motives for their interest in harmful substances in products (question 5). The motive indicated by most participants (89.5%) was the personal quality of life (‘I care for my health and the health of my family.’), followed closely by the three sustainability reasons which were chosen by 79.3–77.6% of the participants (‘I want that future generations will have a good life on earth.’ ‘I care for plants and animals in our environment.’ ‘I care for the environment as the basis of my life.’). Interestingly, ‘I care for plants and animals in our environment’ which is purely an environmental motive was rated as high as the answers with reference to the human being. Compared to Fig. 1 where the health aspects were rated as more important for eight out of eleven product groups, it is interesting to note, that general sustainability motives were considered almost as important as personal quality of life in this question.

The more practical reasons (‘I am interested in chemistry.’ ‘I am interested in the functional principles of chemicals in products.’ ‘I am interested for job reasons.’) were chosen by 24–22.6% of the survey participants. Several respondents (1.8%) added other reasons in their own words (such as ‘*I want to take action against the chemical flood as* ‘*panacea*’.’ ‘*I am concerned about the workers who produce these products.*’ or ‘*The chemical complexity of products is beyond control.*’). Some of them explained their attitude using their own words (such as ‘*I want to minimize unnecessary risks and hazards.*’), while others commented that they would prefer other wordings such as ‘*interested*’ or ‘*curious’* instead of ‘*concerned*.’

On average, four answers were selected. People with no or little chemical expertise chose the first four answers four times more often than the last four more practical motives, whereas people with knowledge of chemistry chose them 2.8 times more often than the last four (*χ* ^2^ = 24.9, *p* < 0.0000).

### Risk communication instruments

**Fig. 2:**
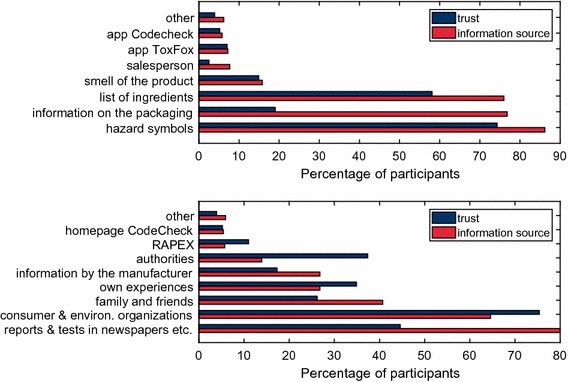
Information sources about harmful substances in products. Red bars show number of information sources indicated to be used by participants directly at the point of sale (above) or elsewhere (below). Blue bars show the confidence in these information sources that the participants reported

Survey participants were asked to select the sources which they use for obtaining information about harmful substances on the products directly and at the point of sale (question 8) or elsewhere (question 9). Furthermore, they could rate their confidence in these information sources (question 10) (Fig. 2).

#### Hazard pictograms, information on the packaging, and list of ingredients are preferred information sources on products

Hazard pictograms (86.2%), information on the packaging (76.8%), and list of ingredients (76.0%) were by far the most frequently chosen options for information that can be obtained at the point of sale (Fig. 2 above). It must be noted here that hazard pictograms and list of ingredients are information sources for mixtures, not for articles (see Box 1). These results obtained in the present survey of ‘best-case’ participants are higher than in the survey with average European participants (66% use safety instructions, 65% warning symbols, 43% packaging) [5].

The fourth most important cue selected by 15.8% of the respondents was the smell of a product which they used to find out whether it would contain harmful substances. In comparison, the participants in the Eurobarometer study selected the smell more frequently as information source (21%) [5].

The smartphone applications were chosen by less than 10% each (ToxFox 7.2%, CodeCheck 5.8%) and twice as frequently by women (16.6%) compared to men (7.7%) (ToxFox *χ* ^2^ = 3.7, *p* < 0.06; CodeCheck *χ* ^2^ = 14.8, *p* < 0.0002). Nearly three quarters (72.6%, *χ* ^2^ = 25.5, *p* < 0.0003) of CodeCheck users are between 20 and 39 years old, while 59.7% (*χ* ^2^ = 20.1, *p* < 0.003) of the ToxFox users are in this age class. Only one person below 20 and only five persons above 60 are using one of these smartphone applications.

On average, each participant indicated three information sources at the point of sale.

One out of ten participants (9.5%) did not know how to find out at the point of sale whether a product contains substances harmful for human health or for the environment. Surprisingly, even persons who indicate to have a good or very good chemical expertise (7%, *χ* ^2^ = 10.2, *p* < 0.002), and persons who work with chemicals or REACH in their job (6.2%, *χ* ^2^ = 6.1, *p* < 0.02) did not know how where to find out more information.

Several participants made additional suggestions which show a thorough understanding of the matter, such as: ‘*The toxicity depends on the dose applied, this is also true for natural substances.’ ‘Substances of very high concern are not declared, hence information requests by consumers according to REACH,*—*but does an answer arrive ever?’ ‘There is no safety, as long as there is no labeling requirement, e.g., for PFCs or NPEO.’* Other additional suggestions show a critical attitude: *‘I am afraid that this can be discerned in few cases only.’ ‘Information on the product often cannot be understood by laypersons.’ ‘I am sure that I am not aware of all harmful substances on all products, although I know that this would be very important.*’ ‘*By implication: if the product is not marketed as safe, it must be assumed to be risky.*’ Others show a high self-assessment: ‘*I make my assessments depending on the purchasing price, the product properties and its origin.’ ‘I make my own measurements.’ ‘I have my own expertise to understand interactions.*’

#### Public media reports and tests are preferred additional information sources

Among the additional information sources which cannot be found at the point of sale, reports and tests in newspapers, radio, and television were the favored options by most participants (80%) (Fig. 2 below). Especially members of environmental organizations preferred test reports in media (85.6%, *χ* ^2^ = 10.2, *p* < 0.002). Members of non-governmental organizations (NGOs) favored information by NGOs more than the average (members of environmental organizations 75.2%, *χ* ^2^ = 21.4, *p* < 0.000, members of consumer organizations 91.9%, *χ* ^2^ = 13.3, *p* < 0.0003, compared to the average of 64.6%). Family members and friends were the third-most important information source named (40.7%), clearly more often than information by manufacturers (26.8%) or information by authorities (13.9%). Students and trainees were the group who indicated friends and family most often (57%, *χ* ^2^ = 13.7, *p* < 0.0003). Persons who are working with chemicals or REACH at their workplace did not choose information by family and friends (26.3%) as often as the rest of the participants (47.7%, *χ* ^2^ = 44, *p* < 0.0000). Even 19.2% (*χ* ^2^ = 50.9, *p* < 0.0000) of people with very good knowledge of chemistry chose family and friends as information source. Interestingly, only 13% of the participants of the Eurobarometer survey indicated family members and friends [5] as information sources. Every second person with very good chemical expertise (51.4%) chose the scientific literature as information source, while 14.4% of people without chemical expertise indicated using the scientific literature (*χ* ^2^ = 95.8, *p* < 0.0000). Own experiences (26.8%) were named nearly twice as often as authorities (13.9%). Women indicated to use their own experience more often (28.8%) than men (22.7%, *χ* ^2^ = 4.7, *p* < 0.03). This percentage was higher in the Eurobarometer study (32%) [5].

Every fifth person (21.8%) who is working with chemicals or REACH at the workplace would ask authorities for more information, compared to only 9.7% of the other respondents (*χ* ^2^ = 28.2, *p* < 0.0000). The CodeCheck homepage was not ticked by many respondents (total 56), but seven times more often by women (49) compared to men (seven) (*χ* ^2^ = 19.0, *p* < 0.0000). The RAPEX database was not chosen frequently (5.7%). Most of the persons who selected RAPEX (70.5%, *χ* ^2^ = 63.6, *p* < 0.0000) are working with chemicals or REACH at their workplace and have an academic degree. We had expected that most people who reported to have a chemical intolerance would know where to find out more information about the ingredients of a product, but this was not significant (7.5% of them did not know where to find out more information, *χ* ^2^ = 1.4, *p* < 0.3). There were even nine (out of 255) persons with chemical intolerance in their families who did not feel that it was necessary to collect further data on products with harmful substances. Some participants (2.8%) chose the option ‘*Dangerous substances are everywhere. I do not need to enquire further’*.

Several respondents (5.9%) indicated additional possibilities where to find more information. Among them were 25 respondents who indicated unspecifically that they would find more information in the ‘*internet*,’ ‘*google*,’ or ‘*in online blogs*’ which left open how they would find trustful information about harmful substances in a specific product. Others gave somewhat more precise information sources such as ‘*Wikipedia*,’ ‘*Safety data sheets*,’ ‘*ECHA*,’ ‘*GIS Code*,’ ‘*Beat the Microbead App*,’ or ‘http://www.cosmeticanalysis.com *’* while again it remains unclear where the respondents find concrete data for a certain product. (Safety data sheets provide information about mixtures, the GIS Code is for building materials and the ECHA homepage provides information about single substances, but not about particular products.) Other notes show again a high self-assessment: ‘*I use my private literature*.’ ‘*I am a chemist*.’ or a high trust in friends with expertise: ‘*I ask friends who are medical doctors or pharmacists.*’ One note summarized the actual situation: ‘*It is difficult to find good information that is correct and easy to understand*.’

#### High confidence in consumer and environmental organizations and in hazard pictograms

Around three quarters of the respondents considered information published by consumer and environmental organizations (75.4%) and the hazard pictograms of the CLP Regulation (74.3%) as trustworthy information (Fig. 2). More than half of the participants (58.1%) trusted the lists of ingredients, mainly people with very good knowledge of chemistry (69.6%) and less people with little chemical knowledge (47.5%, *χ* ^2^ = 31.5, *p* < 0.0000). Less than half of the respondents (44.6%) trusted the test reports in the press, preferentially people without chemical knowledge (52.7%) and less people with very good knowledge of chemistry (28%, *χ* ^2^ = 34.5, *p* < 0.0000). A lot more people with very good knowledge of chemistry (51.9%) indicated to trust the information provided by authorities compared to people without chemical knowledge (27.9%, *χ* ^2^ = 35.7, *p* < 0.0000). The number of people who trusted the information provided by authorities (37.4%) was only slightly larger than the number of people who trusted their own experiences (34.9%). People with chemical intolerance trusted their own experiences more (40.8%) than people without chemical intolerance (32.9%, *χ* ^2^ = 5.2, *p* < 0.03). More people with chemical intolerance (20.4%) trusted the smell of products compared to 13.0% of people without chemical intolerance (*χ* ^2^ = 8.2, *p* < 0.005). Information by manufacturers was indicated as trustworthy by only 17.3%. Most people who indicated to use the ToxFox smartphone application or CodeCheck considered these information sources as trustworthy.

Eighty respondents made some comments about information sources they would trust: They indicated to trust ‘*scientific literature*’(7), ‘*safety data sheets*’(2), ‘*ECHA*’(2), ‘*independent information in the internet*,’ ‘*the World Wide Fund for Nature*,’ ‘*Labels such as the Blue Angel, TÜV, FSC*, etc.,’ ‘*Oekotest*,’ or ‘*my husband*.’ Some respondents mentioned information sources which they did not trust, for example, ‘*the media and NGOs*.’ Other comments showed the critical attitude in general or in respect of the existing legislation: ‘*I do not trust any information 100*%.’ ‘*Smell is only applicable as information source for a few substances and only above a certain concentration*:’ ‘*A residual insecurity remains*.’ ‘*Legislation has loopholes*.’ ‘*Ingredient lists on food packaging are not complete*.’ ‘*I do not always trust authorities or lists of ingredients*.’ ‘*I have no trust because of missing labelling obligations*.’ ‘*The RAPEX database is always out of date*.’ Finally, there were comments by respondents who considered their own expertise to be sufficient: ‘*Due to my profession, I can usually assess this myself*.’

#### Frequently indicated information sources are not always considered to be trustworthy

Consumer and environmental organizations were selected by many (64.6%) and trusted by even more participants (75.4%). In some cases, however, respondents selected information sources, even if they did not trust them (Fig. 2). For example, the general information on the packaging, which was ticked by 76.8% of the participants as information source, was considered as trustworthy by only 19%. Four out of ten respondents (40.7%) indicated to use information provided by friends and family members, although only 26.2% trusted this information source. A third of the people without chemical knowledge (33.8%) trusted friends and family, whereas only 7.5% of people with very good knowledge of chemistry trusted them (*χ* ^2^ = 52.0, *p* < 0.0000). Interestingly, 34.9% of study participants indicated to trust their own experiences while less (26.8%) actually used them as information source. Own experiences (26.8%) were named nearly twice as often as authorities (13.9%) as information sources, but these two information sources were considered as trustworthy by nearly the same number of persons (34.9% own experience, 37.4% authorities).

### Many respondents were not aware of the presence of harmful substances in products

Participants were asked to indicate which product types they considered to be free of harmful substances (question 11). On average, the respondents chose three options.

Many respondents (61.9%) considered untreated food taken from own private gardens and organic food with the EU label (52.8%) as free of harmful substances. More than a third of the respondents (36.1%) assumed that products with an eco-label did not contain harmful substances. Even more (35.7%) believed that natural personal care products were free of harmful substances. Twice as many women believed this (43.7%), compared to men (22.9%) (*χ* ^2^ = 46.8, *p* < 0.0000). Even 20.6% of persons with very good knowledge of chemistry assumed that natural personal care products would not contain harmful substances, while 46.3% of people with very little chemical knowledge believed this (*χ* ^2^ = 43.1, *p* < 0.0000).

Nearly a third of the respondents (29.6%) believed that homeopathic products were free of harmful substances. Even 15.9% of persons who indicated to have good chemical expertise believed that homeopathic products were free of harmful substances, compared to 36.1% of individuals with little knowledge in chemistry (*χ* ^2^ = 28.1, *p* < 0.0000). Nearly every second member in consumer organizations (45.9%, *χ* ^2^ = 28.1, *p* < 0.0000) thought that homeopathic products were free of harmful substances.

One out of four survey participants (25.5%) assumed that natural pharmaceuticals were free of harmful substances. Even 12.6% of persons with very good knowledge of chemistry assumed that natural pharmaceuticals would not contain any harmful substances, while 34.1% of people with very little chemical knowledge believed this (*χ* ^2^ = 37.4, *p* < 0.0000). More than every third student (38.3%) believed that natural pharmaceuticals did not contain any harmful substances, while 21% of people with an academic degree believed this (*χ* ^2^ = 42.8, *p* < 0.0000).

There were 11.0% of the survey participants (15.0% of men and 8.1% of women, *χ* ^2^ = 12.0, *p* < 0.0005) who thought that products without hazard pictograms were free of harmful substances, and 6.7% of the participants assumed that products for children were free of harmful substances.

Furthermore, 4.5% of the respondents assumed that medical devices were free of harmful substances, 7.7% of the men and 2.3% of the women (*χ* ^2^ = 17.2, *p* < 0.0000).

There were even a few participants (1.7%) who assumed that products produced in the European Union would be free of harmful substances, surprisingly most of these people indicated to have good or very good expertise in chemistry.

A fifth of the respondents (20.1%) stated that none of the products were free of harmful substances. Twice as many persons with very good knowledge of chemistry (30.8%) selected this answer, compared to persons with very little chemical knowledge (15.2%, *χ* ^2^ = 21.3, *p* < 0.0000).

Several survey participants (3.6%) made additional suggestions, many of which show that these participants well understood the matter, such as the following: ‘*In general, all products might contain dangerous substances*.’ ‘*Minor amounts of substances harmful for human health are present everywhere*.’ ‘*The dose makes the poison*.’ ‘*Organic does not mean free from…*.’ ‘*There is no 100% safety*.’ ‘*Natural substances can be dangerous for human health*.’ There were again persons who feel very well informed: ‘*I can assess this very well myself due to my profession*.’

### Avoidance is the favored risk reduction strategy

**Fig. 3:**
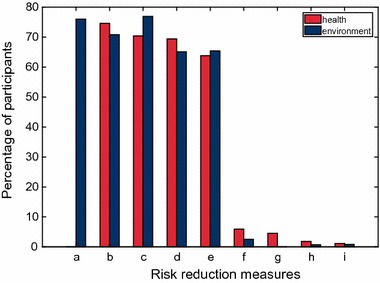
Risk reduction measures indicated by survey participants (personal risks in red, environmental risks in blue). **a** I dispose of the product after use according to instructions. (only in question 13). **b** If possible, I do not buy such products. **c** I use the product as little as possible. **d** I read the description on the packaging carefully. **e** I follow the recommended application and safety instructions on the product and use gloves, for example. **f** I assume that the amounts that I am using are so small that I do not need to be mindful of anything. **g** I trust that the substances will not have negative effects on my health. (only in question 12) **h** Other possibilities:……….. **i** I do not do anything

Risk caused by harmful substances is dependent on exposure. Participants could indicate the measures they use to reduce their personal risks (question 12) or the environmental risk (question 13) (Fig. 3).

There were clearly four preferred options to reduce the personal risk and five to reduce the environmental risks, which were chosen by more than two-thirds of the participants (Fig. 3a–e), while all the other options were of minor importance and stayed below 6% (Fig. 3f–i).

Three quarters (74.6%) indicated that they would not buy such products to reduce their personal risk (b). This percentage was even higher among members of environmental organizations (81.5%, *χ* ^2^ = 11.2, *p* < 0.0000). Slightly less participants (70.8%) indicated that they would not buy such products so as to reduce the environmental risk (b), this is especially true for members of environmental organizations (80%), while 66.6% of non-members chose this option (*χ* ^2^ = 17.1, *p* < 0.0000). Two-thirds of all participants (65.4%) indicated to follow the recommended application and safety instructions for reducing the environmental risks (e). More than half of people with little knowledge in chemistry (57%) selected this option, while 71.5% of persons with very good knowledge in chemistry chose this answer (*χ* ^2^ = 16.9, *p* < 0.003).

Less foremen/business administrators (51.2%, *χ* ^2^ = 13.2, *p* < 0.0003) indicated to follow the instructions for reducing their personal risks compared to an average of 63.8% (e).

There were 2.5% respondents who assumed that the amounts that they were using were so small that they did not need to be mindful of any environmental risk (f). However, no member of consumer organizations believed this. Only 0.8% of the participants thought that it is not necessary to take any measures (i), half of them were people with little self-reported knowledge in chemistry.

### Legislators, consumers, and manufacturers were considered responsible

Participants were asked about their attitude toward the responsibility for the reduction of harmful substances in consumer products in our society (question 14). Nearly all participants (93.9%) considered legislators as responsible for risk reduction. Three quarters of all participants (74.6%) thought that the consumer carries the responsibility. 80% of members of environmental organizations considered consumers as responsible, compared to 71.7% of non-members (*χ* ^2^ = 7.1, *p* < 0.008). Slightly less respondents (71.3%) considered manufacturers and importers as responsible stakeholders. Nearly two-thirds (61.4%) think that environmental and consumer organizations carry the responsibility. Four out of ten (40%) were of the opinion that everybody is responsible, and 35.2% of men and 42.6% of women thought so (*χ* ^2^ = 5.7, *p* < 0.02). Only 0.2% indicated that nobody was responsible.

## Discussion

### Chemical expertise helps, but is not sufficient

The results suggest that even motivated and interested consumers do not generally conceive the complexity of the matter and several of them do not recognize that some of their responses were not correct (e.g., that natural personal care products would not contain hazardous substances). Many respondents could be considered as ‘chemical experts,’ either because they were working with chemicals or because their self-reported knowledge of chemistry was good or very good, and the large majority of participants had an increased interest in their personal health and a pronounced sense of responsibility for the future. Also, some of the comments given by the respondents in the open questions (see answers to questions 5, 8 and 9) proved how qualified some of the survey participants were. They tended to give more correct answers than people with no self-reported knowledge in chemistry, and apparently, people with a chemical expertise have a higher motivation to learn more than people who lack such a basic knowledge (see answers to question 5). However, even persons with good or very good chemical expertise did not always know the real facts. For example, not all of them were aware of harmful substances in consumer products, and a considerable number of them considered natural pharmaceuticals or natural personal care products as free of harmful substances. There were many individuals who did not know how to find out more information about the ingredients of a product, and among them were also persons who indicated to have a good or very good chemical expertise and persons who work with chemicals or REACH in their job.

The low number of smartphone application users is surprising as the questionnaire used for this study could only be filled out online, and hence the respondents were already a selected group of people who are familiar with the internet, which shows that even motivated informed persons are not using these information sources to a considerable extent. This is an important result because this tool is considered to have great chance to contribute to an improved risk communication, e.g., in the case of the right to know according to REACH [36]. Although RAPEX is a database set up by the European Union and designed for the general public, it is apparently not widely known among the interested population.

The data of this survey substantiated how difficult it is for the motivated and interested well-informed consumer to make personal risk decisions. Various comments given by participants reflected the great uncertainty consumers have when they are required to make safety decisions. The scientific community is continuously elucidating new findings about the overwhelming number of chemicals, their fates, effects, and interactions. In many cases, there is not even a scientific consensus in the scientific community. This resulting scientific uncertainty is an essential characteristic in risk assessment, which should also be addressed in proper risk communication, but which does not make it easier to understand for the end-user (e.g., [59, 60]). Furthermore, some harmful substances improve the overall product performance or help to reduce connected health or environmental impacts, so that cost–benefit analyses of certain chemical applications make the risk assessments even more complicated.

In their meta-study, Rowe and Wright described that ‘expert judgement of risk is unlikely to be more veridical than that of non-experts’ [8]. This could be confirmed by the present survey. The question, however, is: Who is an expert? The legal framework and scientific knowledge has become so detailed that hardly anybody can be a true expert for more than certain aspects in a topic. More efforts are needed for the transfer of research findings to decision makers who then decide about communication instruments which a lay audience should be able to understand and use [61].

### Dangerous misconceptions: safety or pseudo safety?

Some of the erroneous assumptions and misconceptions of the respondents must be regarded as dangerous as they thwart a decent safety behavior. This is especially true for persons who assumed that products manufactured in the European Union, products without hazard pictograms, natural pharmaceuticals, natural personal care products, homeopathic products or products with an eco-label would be free of harmful substances. There seems to be a lack of awareness of chemical risks even in motivated informed consumers. This results in a subjective feeling of security, which is actually pseudo safety due to insufficient understanding. Given that three out of four respondents postulate that consumers take over the responsibility for minimizing harmful substances in consumer products, this result is rather alarming. It remains unclear how end-users should bear responsibility and reduce the risk posed by harmful substances as long as they do not have the knowledge to make sufficiently informed choices. Avoidance is the favored risk reduction strategy chosen by the majority of the study participants (see answers b and c in Fig. 3), which is a useful general strategy for consumers. Reading the description and instructions on the packaging (see Fig. 3 answers d and e) is only helpful if this leads to the recommended safety behavior. Reading alone does not reduce risk. Also small doses of very toxic substances can lead to severe deleterious effects. Disposal of the product after use according to instructions (see Fig. 3 answer a) is certainly a good way of reducing environmental exposure, but this is not sufficient for products which are discharged via the waste water after use such as washing and cleaning agents, rinse off personal care products or products directly discharged into the environment such as pesticides.

### Differences between individuals must be taken seriously

The answers given by the participants of this survey show that social and cultural factors like gender, family situation, formation, work experience, or health aspects play important roles for the understanding of and attitudes toward harmful substances in products. Consumers make their personal risk assessments not only on the basis of facts, but their decisions are influenced by ethics, emotions, and social and cultural factors [6]. There were substantial differences between the answers given by various groups, e.g., between men/women, members/non-members of NGOs, people with/without academic degree, or parents/non-parents. Affected individuals such as parents of minor children or women seem to be more interested in certain products groups such as toys or personal care products than the average (see answers to questions 3 and 4). Clear differences in the answers could be seen between people with high or low self-reported knowledge of chemistry. However, there was not a single group who performed consistently. For example, members of NGOs gave more appropriate answers than the average in some cases: most members of consumer organizations believed that small amounts of harmful substances also matter and most members of environmental organizations would reduce environmental exposure by not buying such products to reduce the environmental risk, but, members of NGOs gave more inappropriate answers than the average in other cases: nearly half of the members in consumer organizations thought that homeopathic products were free of harmful substances.

### High confidence in NGOs versus low confidence in manufacturers and authorities

A surprisingly strong bias was observed: Three quarters of the survey participants trusted in information provided by NGOs, while only around a third of them had confidence in authorities and only a sixth trusted manufacturers (Fig. 2). Most survey participants did not choose answers where authorities or manufacturers were explicitly named in the offered response options, although these stakeholders should have the best knowledge of harmful substances in products. This might be interpreted by a lack of confidence in authorities and manufacturers. Another possibility is, that persons who provided these answers presumed a higher effort to actively ask these stakeholders for the desired information and assumed a lower chance to receive useful and understandable information (e.g., [62]). It is also possible that the rather low confidence in authorities and manufacturers is one of the reasons for the high personal interest and private engagement of the study participants. The low appreciation of authorities and manufacturers is in contradiction with the fact, that concrete legally requested information by manufacturers on the products (such as hazard pictograms, ingredient lists, and information on the package) were very welcome by the majority of survey participants as information sources (Fig. 2). Apparently, many survey participants were not aware of the roles of manufacturers and did not consider that authorities establish and enforce such obligations.

The media, environmental and consumer organizations, and even family and friends are preferred information sources before authorities or manufacturers (Fig. 2). Four out of ten survey participants ask family and friends, and every fourth participant trusts them. It is questionable whether family members and friends are better experts on harmful substances in products than other parties. These results might therefore be interpreted as a sign of dissatisfaction with the available information sources and mirror the personal uncertainty.

These data might indicate that the risk communication provided by authorities or the manufacturers does not fulfill the expectations by consumers, not even the well-informed and interested consumers, while environmental and consumer organizations enjoy generally a very good reputation not only by their members, but also by non-members.

### Do legislators and consumers carry the responsibility?

Most interested, informed consumers in the present study considered legislators as responsible for the reduction of harmful substances in products, followed by consumers and in the third place manufacturers. This assignment of responsibility is not in line with the polluter pays principle which is one of the basic principles in environmental policy and which puts the responsibility on the shoulders of manufacturers. The REACH Regulation [34] demands that producers and importers take responsibility for managing chemical risks and prove that their products are safe before they can be placed on the market. Legally defined responsibilities depend on the legal frameworks and product types. In contrast to the REACH Regulation and the polluter pays principle, consumers bear a large part of the responsibility in the application of personal care products [4].

Three out of four survey participants considered consumers as responsible for the reduction of harmful substances in consumer products (see answers to question 14). This result might reflect the high motivation of the ‘best-case’ study participants who are prepared to take over responsibility themselves, and it shows again their high commitment to improve the situation. They apparently seem to be aware of every consumer’s contribution to the overall pollution and to the waste of resources, but they do not realize that they would overburden average consumers with the responsibility for the reduction of harmful substances in products. The data in this survey suggest that also informed consumers are overcharged with the responsibility to make informed choices about harmful chemicals in products.

When consumers in the EU member states were asked about their views, who should ensure the safety of chemical substances [7], 60% responded that manufacturers should have that role, 57% said that the authorities of the European Union and 49% said that the national authorities were responsible. Interestingly, that survey [7] did not offer ‘consumers’ or ‘environmental and consumer organizations’ as potential answers, two options that were chosen by high percentages of the participants in the present survey.

### Study limitations

In this survey, it could not be checked whether persons who selected an information source (Fig. 2) are really using this source in everyday practice for the products they purchase and whether they are also understanding the information appropriately. It cannot be ruled out that the term ‘harmful substance’ was not understood correctly by all participants and that some of them confounded it with ‘harmful products,’ not knowing that many ‘safe products’ contain harmful substances. The survey also does not allow deducing whether the respondents apply risk-mitigation strategies in cases where they know that they handle products containing harmful substances. The sampling of the present study participants was not random but represented a ‘best-case’ selection; however, the self-reported demographic data were not verified, and the age classes of the participants were not equally distributed. The demographic question concerning the educational qualification level offered not more than six potential answers so as to allow the authors a pragmatic analysis of this demographic factor. The study participants who indicated to be members in environmental or consumer organizations did not have to name the organization. Study participants could report whether they considered themselves or family members to have a chemical intolerance according to their own criteria, and it was not differentiated between allergic skin reactions, food intolerance, respiratory diseases, or other health problems.

### Recommendations

The results of this survey can serve as a motivation for new efforts to reassess present approaches for an improved risk communication.

The best way to reduce risk is to keep the residual risk as small as possible by minimizing potential exposure to chemicals identified as harmful via a reduction of the amounts of these chemicals in consumer products [15] or via a reduction of the overall consumption. The most frequent risk-mitigation behavior strategies (avoidance and following the use instructions) indicated by the survey participants (Fig. 3) should therefore be complemented by minimizing the presence of harmful substances in products to achieve a non-toxic environment in the future as planned in the EU [12]. As long as this non-toxic environment is not realized, new efforts are prevalent to improve risk communication about harmful substances in consumer products. Only a small fraction of study participants trusted manufacturers and authorities (Fig. 2). This is alarming because these stakeholders possess the best knowledge about the substances present in commodities and they carry the responsibility according to the present legislation. They could improve their reputation and hence the outreach of their risk communication by more transparent honest information policy and using a language that is understandable to consumers [62]. The important roles friends and family play as highly trusted risk communicators (Fig. 2) reveal the need that the public have access to information in a non-scientific format [61]. We suggest that risk communication should be taught at school so that a basic understanding is part of the general education. Such an early contact with this matter would help to fight against the ignorance and the feeling of uncertainty in average consumers. Risk communication may not be considered exclusively as a top-down transmission of information from experts to the target population, but it must respect the recipients’ needs and capacities [63–65]. For example, according to our study, there does not seem to be a strong need for smartphone applications in this field, while other communication instruments seem to be very useful. Successful risk communication implies a continuous iterative process which analyzes whether and to what extent the intended aims of the measures were reached and what needs to be done to improve the public perception of risk information and the safety behavior [3, 11]. Potential information recipients must be involved in the setup of communication tools in a participatory process [9, 61, 63, 66]. The results in the Eurobarometer study shows the large differences between countries [5]. We recommend to conduct an international study with average consumers who receive special risk communication training of various forms with a subsequent examination of their knowledge and their safety behavior. Results of such a study could contribute to the improvement of efficient risk communication instruments.

## Conclusions

Considering that many consumer products in the EU contain chemicals that are harmful for human health or the environment, it is worrying that even in the present ‘best-case’ subgroups, there are a considerable number of persons who are not aware of this fact and do not have the competence to use the qualified risk communication tools appropriately. Participants in the present study declared to use information sources, even if they do not trust them. Such contradictions compel new efforts to improve risk communication about harmful substances in consumer products. Not only the motivated citizens, but also the general public deserve accurate and accessible information to make the deliberate purchase and handling decisions in their daily routine. They have ‘the right to know’ and ‘the right to comprehend’ [61]. There is a huge gap between: on one side, the growing scientific knowledge available with the complex legislation mechanisms that evolved in the recent decades and on the other side, the basic reasonable expectations of consumers. On the basis of the results of the present survey, we compiled the presumed essential needs that consumers have in respect of harmful substances in products (Box 2).

Consumers have the right to expect that the products they use do not contain chemicals which could pose a threat to the environment and their health and safety. However, before the non-toxic environment becomes real, consumers must be informed and trained to be able to make informed decisions.

### Box 2: Condensed proposal of the basic needs motivated consumers have about harmful substances in products

‘I want to use safe products.’

‘I want to know whether there is a risk for me, my family or the environment.’

‘I want to know whether the risk outweighs the benefit.’

‘I want to receive easily accessible, easily understandable and trustworthy information that is relevant for me.’

‘I want to know how to reduce or avoid risk.’

‘I want to be rewarded if I make all these efforts.’

### Additional files

**Additional file 1.** Survey Questions in German.

**Additional file 2: Table S1.** Age groups of study participants and self-reported knowledge of chemistry.

## Appendix: Text of the survey

Welcome!

Welcome to our survey on *chemicals which are harmful for human health or the environment and used in everyday products* (such as furniture, electronic appliances, plastic products). Our aim is to make suggestions for improving the legal framework. Your answers are valuable contributions for our work. We would be happy if you could answer the questionnaire and forward it to friends so that we will receive a large number of answers in the short time span. The analysis of the answers will be anonymous. In case *you are interested in the results of our study*, please send an e-mail to the address indicated at the end of the questionnaire, and we will send them to you. We will cast lots among these addresses and give away three **vouchers** worth 100 Euros each to be redeemed at the shop of the BUND (friends of the earth Germany) (http://www.bundladen.de).

Prof. Dr. Ursula Klaschka (*The University of Applied Sciences Ulm*) is responsible for the survey, which will be conducted until October 31, 2016.

Questions with circular symbols allow only a single answer, whereas questions with rectangular symbols allow several answers.

Thank you for your participation!

1. Is it of interest to you whether everyday products you are using contain chemicals that are harmful for human health or the environment?
  - ○ Yes, basically always. => 3.
  - ○ This is of interest for me for some products, only. => 3.
  - ○ No. => 2.
2. Under which circumstances might you get interested?
  - $\square \quad$ If I had more time.
  - $\square \quad$ If I had fewer other problems.
  - $\square \quad$ If one of my family members or myself would suffer from chemical intolerance.
  - $\square \quad$ If I knew more about chemistry.
  - $\square \quad$ If I would have to know more about it for job reasons.
  - $\square \quad$ If my family and my friends would be interested.
  - $\square \quad$ I can’t imagine that I would ever be interested.
  - $$\square \quad$$ Other reasons:
  - ⇒ 28
3. Which products are of interest to you **for human health reasons**?
  - $\square \quad$ Personal care products.
  - $$\square \quad$$ Food.
  - $$\square \quad$$ Pesticides.
  - $\square \quad$ Washing and cleansing products.
  - $$\square \quad$$ Electronic appliances.
  - $$\square \quad$$ Furniture.
  - $$\square \quad$$ Textiles.
  - $$\square \quad$$ Toys.
  - $$\square \quad$$ Sports equipment.
  - $\square \quad$ Car care products.
  - $\square \quad$ Building materials (e.g., insulation boards, flooring, sealings).
  - $$\square \quad$$ All.
  - $$\square \quad$$ Other:
4. Which products are of interest to you **for environmental reasons**?
  - $\square \quad$ Personal care products.
  - $$\square \quad$$ Food.
  - $$\square \quad$$ Pharmaceuticals.
  - $$\square \quad$$ Pesticides.
  - $\square \quad$ Washing and cleansing products.
  - $$\square \quad$$ Electronic appliances.
  - $$\square \quad$$ Furniture.
  - $$\square \quad$$ Textiles.
  - $$\square \quad$$ Toys.
  - $$\square \quad$$ Sports equipment.
  - $\square \quad$ Car care products.
  - $\square \quad$ Building materials (e.g., insulation boards, flooring, sealings).
  - $$\square \quad$$ All.
  - $$\square \quad$$ None.
  - $$\square \quad$$ Other:
5. Why are you interested in information on everyday products containing chemicals that are harmful for human health or the environment?
  - $\square \quad$ I care for my health and the health of my family.
  - $\square \quad$ I care for plants and animals in our environment.
  - $\square \quad$ I care for the environment as the basis of my life.
  - $\square \quad$ I want that future generations will have a good life on earth.
  - $\square \quad$ I am interested in chemistry.
  - $\square \quad$ I am interested in the functional principles of chemicals in products.
  - $\square \quad$ I am interested for job reasons.
  - $$\square \quad$$ Other reasons:
6. Do you know, that products you can buy in the EU can contain chemicals that are **harmful for human health**?
  - ○ Yes.
  - ○ No.
  - ○ I am not sure.
7. Do you know, that products you can buy in the EU can contain chemicals that are **harmful for the environment**?
  - ○ Yes.
  - ○ No.
  - ○ I am not sure.
8. How do you find out from **the product itself** whether it contains substances harmful for human health or for the environment?
  - $\square \quad$ Information on the packaging.
  - $\square \quad$ List of ingredients.
  - $\square \quad$ Hazard symbols (pictograms with, for example, a skull, an exclamation mark, or a dead tree and a dead fish) (http://www.umweltbundesamt.de/themen/chemikalien/einstufung-kennzeichnung-von-chemikalien).
  - $\square \quad$ I am using the app ToxFox (http://www.bund.net/toxfox).
  - $\square \quad$ I am using the app CodeCheck (http://www.codecheck.info).
  - $\square \quad$ On the basis of the smell of the product.
  - $\square \quad$ I ask the salesperson.
  - $\square \quad$ I do not know how I could find out about this.
  - $$\square \quad$$ Other:
9. In addition to the information on the product, where and how do you gain **further information** on substances that are harmful for human health and the environment?
  - $\square \quad$ Information by the manufacturer.
  - $\square \quad$ I ask the manufacturer on my own initiative.
  - $\square \quad$ Reports and tests in newspapers, radio, and television.
  - $\square \quad$ Consumer and environmental organizations.
  - $\square \quad$ The European database RAPEX for products which do not comply with the laws (http://www.rapex.eu).
  - $$\square \quad$$ Scientific publications.
  - $\square \quad$ Homepage CodeCheck (http://www.codechek.de).
  - $$\square \quad$$ Authorities.
  - $\square \quad$ Family and friends.
  - $\square \quad$ Own experiences with the product.
  - $\square \quad$ Dangerous substances are everywhere. I do not need to enquire further.
  - $\square \quad$ I do not know where to gain further information.
  - $$\square \quad$$ Other:…..
10. Which information do you **trust**?
  - $\square \quad$ List of ingredients on the product packaging.
  - $\square \quad$ Other information on the product packaging.
  - $\square \quad$ Information by the salesperson.
  - $\square \quad$ Information by the manufacturer.
  - $\square \quad$ Hazard symbols (pictograms with, for example, a skull, an exclamation mark, or a dead tree and a dead fish).
  - $\square \quad$ Reports and tests in newspapers, radio, and television.
  - $\square \quad$ Consumer and environmental organizations.
  - $\square \quad$ The European database RAPEX (http://www.rapex.eu).
  - $\square \quad$ Information by authorities.
  - $\square \quad$ App ToxFox (http://www.bund.net/toxfox).
  - $\square \quad$ App CodeCheck (http://www.codecheck.info).
  - $\square \quad$ Experiences made by family and friends.
  - $$\square \quad$$ Own experiences.
  - $$\square \quad$$ Smell.
  - $$\square \quad$$ None.
  - $$\square \quad$$ Other:…………..
11. Which products do you consider free of substances harmful for human health or the environment?
  - $\square \quad$ Products without any indication on the packaging that these substances are present (such as hazard pictograms).
  - $\square \quad$ Organic food produced according to the EU Regulation for organic food with the EU organic products label.
  - $\square \quad$ Untreated food taken from own private garden.
  - $\square \quad$ Natural personal care products.
  - $$\square \quad$$ Natural pharmaceuticals.
  - $$\square \quad$$ Homeopathic products.
  - $$\square \quad$$ Medical devices.
  - $\square \quad$ Products with an ecolabel, for example. the Blue Angel (http://www.blauer-engel.de).
  - $\square \quad$ Products for children.
  - $\square \quad$ Products which are produced in the EU.
  - $\square \quad$ None of these products.
  - $$\square \quad$$ Others:……
12. How do you reduce **your personal risk** when you use products that contain or could contain substances harmful for human health?
  - $\square \quad$ I read the description on the packaging carefully.
  - $\square \quad$ I follow the recommended application and safety instructions on the product and use gloves. for example.
  - $\square \quad$ I use the product as little as possible.
  - $\square \quad$ I assume that the amounts that I am using are so small that I do not need to be mindful of anything.
  - $\square \quad$ I trust that the substances will not have negative effects on my health.
  - $\square \quad$ If possible, I do not buy such products.
  - $\square \quad$ I do not do anything.
  - $$\square \quad$$ Other possibilities:………..
13. How do you reduce the **risk for the environment** when you use products that contain or could contain substances harmful for the environment?
  - $\square \quad$ I read the description on the packaging carefully.
  - $\square \quad$ I follow the recommended application and safety instructions on the product.
  - $\square \quad$ I use the product as little as possible.
  - $\square \quad$ I dispose of the product after use according to instructions.
  - $\square \quad$ I assume that the amounts that I am using are so small that I do not need to be mindful of anything.
  - $\square \quad$ If possible, I do not buy such products.
  - $\square \quad$ I do not do anything.
  - $$\square \quad$$ Other possibilities:………..
14. Who is **responsible** to reduce the use of substances harmful for human health and the environment in our society according to your opinion?
  - $\square \quad$ Manufacturer and importer.
  - $\square \quad$ Legislator who sets the standards.
  - $\square \quad$ Consumer and environmental organizations.
  - $$\square \quad$$ Consumers.
  - $$\square \quad$$ Nobody.
  - $$\square \quad$$ Everybody
    Questions 15.–27. (skipped in this analysis)
    General statistical information about your person.
15. Are you male or female?
  - ○ Male.
  - ○ Female.
16. What is your age?
  - ○ Below 20.
  - ○ 20–29.
  - ○ 30–39.
  - ○ 40–49.
  - ○ 50–59.
  - ○ 60–69.
  - ○ 70 or older.
17. Do you have children who are less than 18 years old?
  - ○ Yes.
  - ○ No.
18. What is your highest educational qualification?
  - ○ Presently at school.
  - ○ Presently student or in job training.
  - ○ Completed vocational training/apprenticeship.
  - ○ Foreman/business administrator.
  - ○ University degree/Ph.D.
  - ○ Other:……
19. How do you assess your knowledge of chemistry?
  - ○ None/little.
  - ○ Good.
  - ○ Very good.
20. Are you dealing with chemicals or REACH at your workplace?
  - ○ Yes.
  - ○ No.
21. Are you member of an environmental organization?
  - ○ Yes.
  - ○ No.
22. Are you member of a consumer organization?
  - ○ Yes.
  - ○ No.
23. Are you an EU citizen?
  - ○ Yes.
  - ○ No.
24. Do you or does any of your family members suffer from chemical intolerance?
  - ○ Yes.
  - ○ No.
  - ➩ Infobox
    Are you** interested in the results of our study**? We are happy to send them to you by e-mail. You can also win one of three vouchers worth 100 Euros each to be redeemed at the shop of the BUND (friends of the earth Germany) (www.bundladen.de). We want to guarantee that your answers will stay anonymous, therefore please send us an E-mail with the word ‘Verlosung’ (tombola) in the subject heading to the following address: REACH-Umfrage-2016@hs-ulm.de.
25. Do you have any comments or notes?

………

Do you want to know more? Here are the **essentials in brief**.

Lots of everyday products contain **dangerous substances**. The **hazard pictograms** and safety phrases on the containers of paints, varnishes, and washing and cleansing products depict the dangers of such a mixture of chemicals (http://www.umweltbundesamt.de/themen/chemikalien/einstufung-kennzeichnung-von-chemikalien). There are also recommendations for safe handling on the container which help you to reduce your personal risk when dealing with the product and to avoid damage to the environment. Ingredients are listed on the containers/packaging of personal care products and washing and cleansing products. In contrast to that, there is no legal obligation that other products such as electronic appliances, plastic products, or furniture have to be labeled with regard to their content of substances harmful to human health or the environment.

You have the right to ask manufacturers of objects, such as electronic appliances, plastic products, or furniture, whether ‘substances of very high concern’ are present in a specific product above a certain concentration (**REACH right to know**). These substances are, for example, carcinogenic, mutagenic, toxic for reproduction, or very critical for the environment. Examples are certain plasticizers for plastic materials (phthalates), cadmium compounds, or arsenic compounds. A list of these ‘substances of very high concern’ is compiled in the framework of the European chemical legislation REACH Regulation (*Registration, Evaluation and Authorization of Chemicals*). This list is extended regularly and available on the internet under, for example, [http://www.reach-clp-biozid-helpdesk.de/de/REACH/Kandidatenliste/Kandidatenliste.html]. It does not need to be written on a product whether it contains these substances. Instead you may send an inquiry for a certain product to the manufacturer or the distributor which he must answer within 45 days, in case such a substance is present in the product above the threshold of 0.1 weight %. You find a sample letter (http://www.reach-info.de/auskunftsrecht.htm) and an online form (http://www.reach-info.de/verbraucheranfrage.htm) on the homepage of the German Environment Agency (UBA). UBA is currently working on a smartphone app ‘Scan4Chem’ which will facilitate the information request. The app ToxFox by the BUND (Friends of the Earth Germany) will consider substances of very high concern in addition to endocrine substances in future.

One of the aims of the **right to know** by the European Regulation on chemicals (REACH) is that manufacturers replace these substances by less-harmful substances in the long term. Please find further information under (http://www.reach-info.de/svhc.htm) or (http://www.reach-clp-biozid-helpdesk.de/de/REACH/SVHC-Roadmap/Roadmap.html).
