# Supplementary material for: Interested consumers’ awareness of harmful chemicals in everyday products
Source: Environ Sci Eur. 2017 Nov 21;29(1):29. doi: 10.1186/s12302-017-0127-8 (PMC5698398; doi:10.1186/s12302-017-0127-8)
Supplement: Supplementary file 1 — Additional file 1. Survey Questions in German. [file 12302_2017_127_MOESM1_ESM.docx]

Herzlich willkommen zu unserer Umfrage zum Thema **gesundheits- und umweltgefährdende Chemikalien in Alltagsprodukten** (wie z.B. Möbel, Elektronikgeräte, Kunststoffprodukte). Wir möchten Vorschläge zur Verbesserung der europäischen Regelungen erarbeiten, und dazu können Sie mit Ihren Antworten einen wichtigen Beitrag leisten. Wir würden uns freuen, wenn Sie diesen Fragebogen beantworten und wenn möglich an Freunde und Bekannte **weiterleiten** könnten, damit wir in der kurzen Zeit möglichst viele Antworten erhalten. Die Auswertung erfolgt anonym. Falls Sie **Interesse an den Ergebnissen der Studie** haben, schicken Sie bitte am Ende der Umfrage eine Mail an die dort angegeben Adresse, dann schicken wir sie Ihnen gerne zu. Unter diesen Adressen werden drei **Geldgutscheine** im Werte von je 100 Euro für den BUND-Laden ([www.bundladen.de](http://www.bundladen.de)) ausgelost

Die Umfrage wird unter Federführung von Prof. Dr. Ursula Klaschka (**Hochschule Ulm) in Kooperation mit dem Umweltbundesamt** bis zum 31.10.2016 durchgeführt.

Im Folgenden ist bei Fragen mit kreisförmigen Symbolen nur eine Antwort möglich, während rechteckige Symbole eine Mehrfachauswahl erlauben.

Wir danken für Ihre Teilnahme.

1. Interessieren Sie sich dafür, ob Alltagsprodukte, die Sie verwenden, gesundheits- und umweltgefährdende Chemikalien enthalten?

- Ja, grundsätzlich immer =>3.
- Es interessiert mich nur bei manchen Produkten => 3.
- Nein. =>2.

2. Unter welchen Umständen könnten Sie sich dafür interessieren?

- Wenn ich mehr Zeit hätte.
- Wenn ich weniger andere Sorgen hätte.
- Wenn eines meiner Familienmitglieder oder ich selbst eine Chemikalienunverträglichkeit hätte.
- Wenn ich mehr über Chemie wüsste.
- Wenn ich aus beruflichen Gründen mehr wissen müsste.
- Wenn meine Freunde und meine Familie sich dafür interessieren würden.
- Ich kann mir nicht vorstellen, dass mich das jemals interessieren wird.
- Andere Umstände:…..
- *28.*

3. Bei welchen Produkten interessieren Sie sich aus **gesundheitlichen** Gründen für die Inhaltsstoffe?

- Körperpflegemittel
- Nahrungsmittel
- Schädlingsbekämpfungsmittel
- Wasch- und Reinigungsmittel
- Elektronikgeräte
- Möbel
- Textilien
- Spielzeug
- Sportgeräte
- Autopflegeprodukte
- Bauprodukte (z.B. Dämmplatten, Fußbodenbeläge, Dichtungen)
- Alle
- Andere:……
- 4.

4. Bei welchen Produkten interessieren Sie sich aus **Umweltschutzgründen** für die Inhaltsstoffe?

- Körperpflegemittel
- Nahrungsmittel
- Arzneimittel
- Schädlingsbekämpfungsmittel
- Wasch- und Reinigungsmittel
- Elektronikgeräte
- Möbel
- Textilien
- Spielzeug
- Sportgeräte
- Autopflegeprodukte
- Bauprodukte (z.B. Dämmplatten, Fußbodenbeläge, Dichtungen)
- Alle
- Keine
- Andere:…..
- 5.

5. **Aus welchen Gründen** interessieren Sie sich dafür, ob die Alltagsprodukte, die Sie verwenden, gesundheits- oder umweltgefährdende Chemikalien enthalten?

- Ich bin besorgt um meine Gesundheit und die meiner Familie
- Ich bin besorgt um Pflanzen und Tiere in unserer Umwelt.
- Ich bin besorgt um die Umwelt als meine Lebensgrundlage.
- Ich möchte, dass auch nachfolgende Generationen auf der Erde gut leben können.
- Ich interessiere mich für Chemie.
- Ich interessiere mich für die Funktionsweise dieser Stoffe in Produkten.
- Ich interessiere mich aus beruflichen Gründen
- Andere Gründe:…….
- 6.

6. Ist Ihnen bekannt, dass Produkte, die Sie in der EU kaufen können, **gesundheitsgefährdende** Chemikalien enthalten können?

- Ja
- Nein
- Weiß ich nicht
- 7.

7. Ist Ihnen bekannt, dass Produkte, die Sie in der EU kaufen können, **umweltgefährdende** Chemikalien enthalten können?

- Ja
- Nein
- Weiß ich nicht
- 8.

8. Wie erkennen Sie  **direkt am Produkt,** ob gesundheits- und/oder umweltgefährdende Chemikalien enthalten sind?

- Informationen auf der Verpackung
- Inhaltsstoffliste
- Gefahrensymbole (Piktogramme mit Totenkopf, Ausrufezeichen, totem Fisch und Baum…) (http://www.umweltbundesamt.de/themen/chemikalien/einstufung-kennzeichnung-von-chemikalien)
- Ich nutze die App ToxFox ([www.bund.net/toxfox)](http://www.bund.net/toxfox)).
- Ich nutze die App CodeCeck [(www.codecheck.info](http://(www.codecheck.info)).
- Ich frage beim Verkäufer/der Verkäuferin nach.
- anhand des Geruchs
- Ich weiß nicht, woran ich das erkennen kann.
- Anderes:…….
- 9.

9. Woher haben oder beziehen Sie zusätzlich zu den Informationen auf dem Produkt Ihre **Kenntnisse**, ob Produkte gesundheits- oder umweltgefährdende Chemikalien enthalten?

- Aussagen des Herstellers
- Ich frage selbst beim Hersteller nach.
- Berichte und Tests in Zeitung, Radio und Fernsehen
- Verbraucher- und Umweltschutzinstitutionen
- europäische Datenbank Rapex über Produkte, die den geltenden Gesetzen nicht entsprechen ([www.rapex.eu](http://www.rapex.eu))
- wissenschaftliche Publikationen
- Homepage CodeCheck [(www.codecheck.info](http://(www.codecheck.info))
- Behörden
- Familienmitglieder und Freunde
- eigene Erfahrungen mit dem Produkt
- Gefahrstoffe sind überall, da brauche ich mich nicht weiter zu erkundigen.
- Ich weiß nicht, wo ich solche Informationen bekommen kann.
- Anderes:…….
- 10.

10. Welchen Informationen **vertrauen** Sie?

- Inhaltsstoffliste auf der Produktverpackung
- Andere Informationen auf der Produktverpackung
- Aussagen des Verkäufers / der Verkäuferin
- Angaben des Herstellers
- Gefahrensymbole (Piktogramme mit Totenkopf, Ausrufezeichen, totem Fisch und Baum…)
- Presse-, Radio- und Fernsehberichte und Tests
- Informationen von Verbraucher- und Umweltschutzinstitutionen
- der europäischen Produktdatenbank Rapex (www.rapex.eu)
- Einschätzungen und Informationen von Behörden
- App ToxFox ([www.bund.net/toxfox)](http://www.bund.net/toxfox)).
- App CodeCheck [(www.codecheck.info](http://(www.codecheck.info))
- den Erfahrungen von Freunden und Familienmitgliedern
- den eigenen Erfahrungen
- dem Geruch
- keinen
- andere:….
- 11.

11. Bei welchen Produkten gehen Sie davon aus, dass **keine** gesundheits- und umweltgefährdenden Stoffe enthalten sind?

- Bei Produkten, auf denen keine Hinweise auf solche Stoffe stehen (z.B. keine Gefahren-Piktogramme)
- Bei Bio-Lebensmitteln, z.B. nach der EU-Ökoverordnung mit dem Bio-Label.
- Bei naturbelassenen Lebensmitteln, z.B. aus dem eigenen Garten
- Bei Naturkosmetik
- Bei Naturheilmitteln
- Bei homöopathischen Produkten
- Bei Medizinprodukten
- Bei Produkten mit einem Umweltsiegel, wie z.B. dem Blauen Engel ([www.blauer-engel.de](http://www.blauer-engel.de))
- Bei Kinderprodukten
- Bei Produkten, die in der EU hergestellt wurden
- Bei keinem dieser Produkte
- Andere:…….
- 12.

12. Wie reduzieren Sie **Ihr persönliches Risiko** beim Umgang mit Produkten, die gesundheitsgefährdende Stoffe enthalten oder enthalten können?

- Ich lese die Informationen auf der Verpackung aufmerksam.
- Ich richte mich nach den Anwendungsempfehlungen und Sicherheitsratschlägen auf der Verpackung und verwende z.B. Handschuhe.
- Ich verwende das Produkt so wenig wie möglich.
- Ich gehe davon aus, dass die Mengen, die ich verwende, so gering sind, dass ich nichts beachten muss.
- Ich vertraue darauf, dass die Stoffe keine negativen Auswirkungen auf meine Gesundheit haben werden.
- Ich kaufe solche Produkte nicht.
- Gar nicht.
- Andere Möglichkeiten:…..

=>13.

13. Wie reduzieren Sie das **Risiko für die Umwelt** beim Umgang mit Produkten, die umweltgefährdende Stoffe enthalten?

- Ich lese die Informationen auf der Verpackung aufmerksam.
- Ich richte mich nach den Anwendungsempfehlungen und Sicherheitsratschlägen auf der Verpackung.
- Ich verwende das Produkt so wenig wie möglich.
- Ich entsorge das Produkt vorschriftsgemäß.
- Ich gehe davon aus, dass die Mengen, die ich verwende, so gering sind, dass ich nichts beachten muss.
- Wenn möglich, kaufe ich solche Produkte nicht.
- Gar nicht.
- Andere Möglichkeiten:
- 14

14. Wer ist Ihrer Meinung nach dafür **verantwortlich**, dass die Verwendung gesundheits- und umweltgefährdender Stoffe in unserer Gesellschaft minimiert wird?

- Hersteller oder Importeure
- Der Gesetzgeber, der entsprechende Vorgaben macht
- Verbraucher- und Umweltverbände
- Die Verbraucher/-innen.
- Niemand
- Jede/-r
- => 15

15. -27 (in dieser Auswertung übersprungen)

**Angaben zur Person**

28. Sind Sie männlich oder weiblich?

- Männlich
- weiblich

29. Wie alt sind Sie?

- unter 20
- 20-29
- 30-39
- 40-49
- 50-59
- 60-69
- 70 oder älter

30. Haben Sie Kinder, die unter 18 Jahre alt sind?

- Ja
- nein

31. Was ist Ihr höchster Bildungsabschluss?

- Derzeit Schüler
- Derzeit im Studium oder in der Ausbildung
- abgeschlossene Ausbildung /Lehre
- abgeschlossene Ausbildung zum Meister / Fachwirt
- abgeschlossenes Hochschulstudium/Promotion
- sonstiges

32. Wie schätzen Sie Ihre Kenntnisse von Chemie ein?

- Keine bis geringe
- Gute
- sehr gute

33. Haben Sie beruflich mit Chemikalien und REACH zu tun?

- Ja
- Nein

34. Sind Sie Mitglied in einer Umweltorganisation?

- Ja
- Nein

35. Sind Sie Mitglied in einer Verbraucherschutzorganisation?

- Ja
- Nein

36. Sind Sie EU-Bürger?

- Ja
- Nein

37. Ist bei Ihnen oder in Ihrer Familie eine Unverträglichkeit von Chemikalien bekannt?

- Ja
- Nein

**Sind Sie an den Ergebnissen dieser Umfrage interessiert?** Dann schicken wir sie Ihnen gerne zu. Damit nehmen Sie auch an der Verlosung der drei Gutschein im Wert von **100 Euro** beim **BUND-Laden** ([www.bundladen.de](http://www.bundladen.de)) teil. Um die Anonymität Ihrer Antworten zu gewährleisten, schicken Sie uns dazu bitte eine E-Mail mit dem **Betreff „Verlosung“** an folgende Adresse: **REACH-Umfrage-2016@hs-ulm.d**e.

38. Haben Sie **Kommentare und Anmerkungen**? .......

Wollen Sie mehr zu dem Thema wissen? Hier ist **in Kürze das Wesentliche** zusammengefasst:

In sehr vielen Alltagsprodukten sind **Gefahrstoffe** enthalten. Bei Farben, Lacken, Wasch- und Reinigungsmitteln können Sie anhand der **Gefahrenpiktogramme**

und Sicherheitshinweise auf den Verpackungen schnell erkennen, welche Gefahren von der Stoffmischung ausgehen ([www.umweltbundesamt.de/themen/chemikalien/einstufung-kennzeichnung-von-chemikalien](http://www.umweltbundesamt.de/themen/chemikalien/einstufung-kennzeichnung-von-chemikalien)). Es sind dort auch Empfehlungen aufgedruckt, wie Sie sich verhalten können, um Ihr persönliches Risiko beim Umgang mit dem Produkt möglichst gering zu halten und die Umwelt nicht zu gefährden. Bei Körperpflege- und Wasch- und Reinigungsmitteln werden die Inhaltsstoffe direkt auf der Verpackung angegeben. Auf anderen Produkten, wie z.B. Elektronikgeräten, Kunststoffartikeln oder Möbeln sind Angaben auf den Verpackungen, ob gesundheits- oder umweltgefährdende Stoffe enthalten sind, nicht vorgeschrieben.

Bei Gegenständen wie Elektronikgeräten, Kunststoffartikeln und Möbeln haben Sie das Recht, beim Hersteller zu erfragen, ob sogenannte besonders besorgniserregende Stoffe über einer bestimmten Menge enthalten sind („**REACH-Auskunftsrecht**“). Diese Stoffe sind z.B. krebserzeugend, erbgutverändernd, fortpflanzungsschädigend oder besonders gefährlich für die Umwelt. Beispiele sind bestimmte Weichmacher für Kunststoffe (Phthalate), Cadmiumverbindungen oder Arsenverbindungen. Im Rahmen der europäischen Chemikaliengesetzgebung (REACH-Verordnung, *Registration, Evaluation and Authorization of Chemicals*) wird eine Liste dieser sogenannten „besonders besorgniserregenden Stoffe“ erstellt. Diese Liste wird regelmäßig erweitert und ist im Internet z.B. unter [http://www.reach-clp-biozid-helpdesk.de/de/REACH/Kandidatenliste/Kandidatenliste.html] zu finden. Bei Gegenständen muss nicht auf dem Produkt angegeben werden, ob diese Stoffe enthalten sind. Sie haben aber das Recht, eine Anfrage für das jeweilige Produkt an den Lieferanten oder Hersteller des Produkts zu schicken. Dieser muss innerhalb von 45 Tagen antworten, wenn ein besonders besorgniserregender Stoff im Produkt über 0,1 Gewichts-% enthalten ist. Auf der Homepage des Umweltbundesamtes gibt es dazu einen **Musterbrief** und ein **Online-Formular** ([www.reach-info.de/auskunftsrecht.htm](http://www.reach-info.de/auskunftsrecht.htm)). Unter der Federführung des Umweltbundesamtes ist eine App „**Scan4Chem**“ in Arbeit, die Abfragen noch weiter erleichtern wird. Auch die App des BUND (Bund für Umwelt und Naturschutz) **ToxFox** wird in Zukunft neben den hormonartig wirkenden Stoffen auch die besonders besorgniserregenden Stoffe berücksichtigen.

Die europäische Verordnung zu Chemikalien (REACH) verfolgt mit dem Auskunftsrecht unter anderem das Ziel, dass Hersteller diese Stoffe langfristig durch weniger gefährliche ersetzen. Weitere Infos unter ([www.reach-info.de/svhc.htm](http://www.reach-info.de/svhc.htm)) oder ([www.reach-clp-biozid-helpdesk.de/de/REACH/SVHC-Roadmap/Roadmap.html](http://www.reach-clp-biozid-helpdesk.de/de/REACH/SVHC-Roadmap/Roadmap.html) ).
