# Supplementary material for: Interested consumers’ awareness of harmful chemicals in everyday products
Source: Environ Sci Eur. 2017 Nov 21;29(1):29. doi: 10.1186/s12302-017-0127-8 (PMC5698398; doi:10.1186/s12302-017-0127-8)
Supplement: Supplementary file 2 — Additional file 2: Table S1. Age groups of study participants and self-reported knowledge of chemistry. [file 12302_2017_127_MOESM2_ESM.docx]

Additional file

*Table S1:
Age groups of study participants and self-reported knowledge of chemistry.*

|  | < 20 | 20-29 | 30-39 | 40-49 | 50-59 | 60-69 | >70 | Sum |
| --- | --- | --- | --- | --- | --- | --- | --- | --- |
| No or little knowledge in chemistry | 16%  Female: 16%  Male: 0% | 33,2%  Female: 20.5%  Male: 12.7% | 32,4%  Female: 23.8%  Male: 8.6% | 43,5%  Female: 32.4%  Male: 11.2% | 44,2%  Female: 31.8%  Male: 12.4% | 47,4%  Female: 25.9%  Male: 21.6% | 51,9%  Female: 20.4%  Male: 31.5% | 402  (39%) |
| good knowledge in chemistry | 80%  Female: 52%  Male: 28% | 44,1%  Female: 28.2%  Male: 15.9% | 39,3%  Female: 24.2%  Male: 15.2% | 37,6%  Female: 23.5%  Male: 14.1% | 39,8%  Female: 23.9%  Male: 15.9% | 37,1%  Female: 11.2%  Male: 25.9% | 25,9%  Female: 3.7%  Male: 22.2% | 414  (40.2%) |
| Very good knowledge in chemistry | 4%  Female: 0%  Male: 4% | 22,7%  Female: 14.1%  Male: 8.6% | 28,3%  Female: 11.5%  Male: 16.8% | 18,8%  Female: 9.4%  Male: 9.4% | 15,9%  Female: 9%  Male: 7% | 15,5%  Female: 3.4%  Male: 12.1% | 22,2%  Female: 1.9%  Male: 20.4% | 214  (20.8%) |
| Sum | 25  (2.4 %) | 220  (21.4%) | 244  (23.7%) | 170  (16.5%) | 201  (19.5%) | 116  (11.3%) | 54  (5,2%) | 1030  (100%) |
